# Mitochondrial DNA data indicate an introduction through Mainland Southeast Asia for Australian dingoes and Polynesian domestic dogs

**DOI:** 10.1098/rspb.2011.1395

**Published:** 2011-09-07

**Authors:** Mattias C. R. Oskarsson, Cornelya F. C. Klütsch, Ukadej Boonyaprakob, Alan Wilton, Yuichi Tanabe, Peter Savolainen

**Affiliations:** 1 Science for Life Laboratory, Department of Gene Technology, KTH-Royal Institute of Technology, 106 91 Stockholm, Sweden; 2 Department of Physiology and Biochemistry, Faculty of Veterinary Medicine, Kasetsart University, Bangkok, Thailand; 3 Ramaciotti Centre for Gene Function Analysis, School of Biotechnology and Biomolecular Sciences, University of New South Wales, Sydney, New South Wales 2052, Australia; 4 School of Veterinary Medicine, Azabu University, Sagamihara 229-8501, Japan

**Keywords:** mitochondrial DNA, dingo, Polynesia, Australia, dog, haplotype

## Abstract

In the late stages of the global dispersal of dogs, dingoes appear in the Australian archaeological record 3500 years BP, and dogs were one of three domesticates brought with the colonization of Polynesia, but the introduction routes to this region remain unknown. This also relates to questions about human history, such as to what extent the Polynesian culture was introduced with the Austronesian expansion from Taiwan or adopted en route, and whether pre-Neolithic Australia was culturally influenced by the surrounding Neolithic world. We investigate these questions by mapping the distribution of the mtDNA founder haplotypes for dingoes (A29) and ancient Polynesian dogs (Arc1 and Arc2) in samples across Southern East Asia (*n* = 424) and Island Southeast Asia (*n* = 219). All three haplotypes were found in South China, Mainland Southeast Asia and Indonesia but absent in Taiwan and the Philippines, and the mtDNA diversity among dingoes indicates an introduction to Australia 4600–18 300 years BP. These results suggest that Australian dingoes and Polynesian dogs originate from dogs introduced to Indonesia via Mainland Southeast Asia before the Neolithic, and not from Taiwan together with the Austronesian expansion. This underscores the complex origins of Polynesian culture and the isolation from Neolithic influence of the pre-Neolithic Australian culture.

## 1. Introduction

The domestic dog is unique in that it was the only domestic animal accompanying humans to every continent in ancient times. The dog has been present as the sole domestic animal in ancient Australia, in the form of feral dingoes, since at least 3500 years BP [1,2], and was introduced to Polynesia together with chickens and pigs when this region was colonized by human settlers starting approximately 3000 years BP [3–5]. However, the introduction routes and ultimate origin for these dogs are not known. Therefore, how dogs initially spread from mainland Asia to this part of the world remains to be investigated.

This dispersal of dogs is also linked to the human history of the region, and may contribute knowledge about, for example, the geographical origins of the Polynesian population and its Neolithic culture, and the extent of contact between the pre-Neolithic cultures of Australia with the surrounding world.

Australia and New Guinea were first colonized by hunter–gatherers approximately 50 000 years BP [6], which by 30 000 years BP had reached as far into Near Oceania as the Bismarck Archipelago and the westernmost Solomon Islands [3,7]. However, the islands further east remained without human presence until the arrival of the Neolithic, which reached western Polynesia by 3000 years BP, and all the way to eastern Polynesia approximately 1400 years BP [8].

The domestication of rice in the Yangtze valley, which occurred at least 8500 years BP [5], led to a spread of agriculture and Neolithic culture that reached Southeast Asia between 4500 and 3500 years BP, and Taiwan by at least 5500 years BP [5]. By approximately 3500 years BP, an archaeologically defined Neolithic cultural complex, called Lapita [4], appeared in Near Oceania, and within 500 years a material culture stemming from Lapita had spread east into the previously unpopulated Polynesia, finally reaching New Zealand by AD 1250 [5]. This dispersal is obviously linked to the spread of the ancestors of the Polynesians and of the Austronesian languages, and represents one of the most extensive geographical expansions of a human population in history. The Polynesian material culture consisted of a number of Neolithic items, including the domestic dog, pig and chicken. The ultimate origin of the Polynesians and their culture, and the related Lapita cultural complex, is debated.

Contrastingly, Australia has remained largely isolated, keeping the pre-Neolithic culture with virtually no influence from external sources [6]. An exception is the dingo, a feral dog appearing in the Australian archaeological record at 3500 years BP [1,2]. How the dingo, as the single item of possibly Neolithic origin, arrived to Australia is therefore an enigma.

According to studies of mitochondrial DNA (mtDNA), the domestic dog probably originates from South China 10 000–16 000 years BP [9]. In the region studied here (South China, Mainland and Island Southeast Asia, Australasia and Polynesia), the archaeological record for dogs is generally sparse, but a spread of dogs into the region approximately in parallel with the spread of Neolithic culture is clearly indicated. Archaeological evidence shows that domestic dogs were established along the Gulf of Thailand at 4000 years BP [10], remains from northern Moluccas are dated to 3300 years BP [5,11], and the Yüan-shan culture of northern Taiwan probably possessed dogs from 4500 years BP [11]. The earliest archaeological evidence for dingoes in Australia comes from Nullarbor plains in the southern part of the continent and has been dated to 3500 years BP [1,2]. It is noteworthy that at approximately the same time, 3500 years BP, pigs appear in the archaeological record of eastern Indonesia [12]. In Polynesia, there is indisputable evidence of dogs by 2000 years BP [13].

The origin of the people and culture in Polynesia has been heavily debated, and several different models have been proposed based on archaeological, linguistic, cultural and genetic evidence, as summarized in the study of Hurles *et al*. [14]. The ‘express train model’ [15,16] suggests a rapid spread of farmers from Taiwan approximately 5000 years BP, with little genetic mixing and cultural exchange between farmers and indigenous Melanesians at the eastward spread through Indonesia. Thus, the model suggests that genes, culture and the Austronesian language largely spread as a single entity to Polynesia from Taiwan, and that the Neolithic Lapita cultural complex, including the domestic dog, chicken and pig [16], originated in Taiwan and was introduced as one package, with little supplement on the way.

A model at the other extreme, called the ‘entangled bank model’, suggests a local development of Lapita through a complex network of interactions between southeast Asians, Melanesians and Polynesians over a long time, and not from a Taiwanese farmer expansion [17,18]. A third model, the ‘intrusion–innovation–integration (triple I) model’, proposes that some elements of Lapita already existed in Near Oceania, some were additions from outside sources (e.g. Taiwan), some developed in Near Oceania, and finally a composite culture moved into Polynesia [19].

In the archaeological record, pottery is absent in pre-Lapita archaeological assemblages in Near Oceania [4], and the Lapita pottery is most probably traced to a precursor pottery of the Dapenkeng culture in Taiwan 5500–5000 years BP [4,5,20]. The Austronesian language family is one of the largest and most widespread in the world, spoken from Madagascar to Easter Island [21]. The vast majority of Austronesian language subgroups are spoken only on Taiwan, and the root for the Austronesian language tree falls in Taiwan [22,23]. Human genetic evidence indicates a primarily Indonesian–Melanesian origin for both the Polynesian mtDNA and the Y chromosome gene pools, and only a minor contribution from Taiwan [24–32]. Thus, archaeological and linguistic evidence indicate a Taiwanese origin for the Polynesian culture, while genetic evidence points to a largely Melanesian ancestry for the Polynesian people.

The prehistory of Australia seems unaffected by the Neolithic farming expansion, except for the introduction of the (possibly Neolithic-related) dingo. This is also evident from genetic studies of Aboriginal Australians: analyses of mtDNA, Y chromosome and whole genome genetic diversity indicate long genetic isolation [33,34].

For dogs, Australian dingoes and archaeological samples of ancient Polynesian dogs have been studied for mtDNA, but the introduction route to Australia and Polynesia is not known, because of a lack of data from Mainland and Island Southeast Asia. Therefore, how and why the dingo arrived in the otherwise isolated Australia is an enigma, and it remains unknown where the dogs in Polynesia had their ultimate origin before being included in the Lapita-derived cultural complex.

The study of mtDNA among Australian dingoes and Polynesian dogs showed that archaeological samples of pre-European dog from across Polynesia (the Cook Islands, Hawaii and New Zealand) carried only two haplotypes: Arc1 and Arc2 [35]. It also showed that Australian dingoes carried only haplotype A29 or haplotypes differing from A29 by a single mutation, indicating that the dingo population was founded from a small number of dogs carrying a single mtDNA haplotype (A29). Importantly, all three haplotypes are typical for East Asian dogs: Arc2 and A29 are absent and Arc1 rarely found west of the Himalayas [9]. Furthermore, two New Guinea singing dogs (NGSDs; a feral dog from the New Guinean highlands, close in morphology and behaviour to Australian dingoes but clearly distinguishable [36]) were shown to carry haplotypes A29 and A79 (which differs by one substitution from A29). Since A29 is also found among East Asian dogs, an origin from domestic dogs seems clear for these two wild populations. Based on the mtDNA diversity, the time of arrival of dingoes to Australia was estimated to approximately 5000 years BP, and possibly up to 10 000 years BP [35], indicating an earlier date than the 3500 BP suggested by the archaeological evidence.

We wanted to investigate the origin and route of introduction of Polynesian domestic dogs and the feral Australian dingoes and NGSDs, in order to establish how dogs populated this part of the world and which human cultures may have been involved in these migrations. In this study, we therefore analysed mtDNA for 305 samples of domestic dog from Mainland and Island Southeast Asia to investigate the distribution of the two Polynesian haplotypes, and the Australian dingo and NGSD founder haplotype.

## 2. Material and methods

### (a) Samples

**Figure 1.**
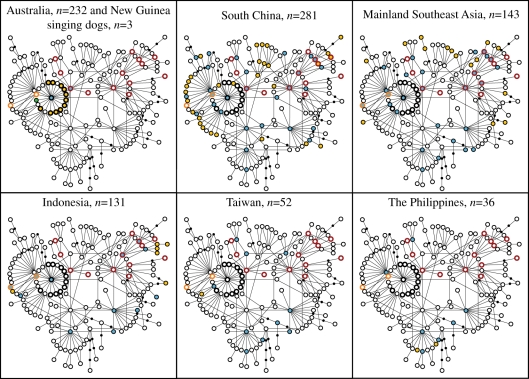
Minimum spanning network showing the genetic relationships of haplotypes in the major dog haplogroup, clade A, and their representation in geographical regions. Haplotypes, found among the 909 dogs and dingoes specifically studied here, and in a global sample of 1224 dogs [9], are represented by circles; lines represent one mutational step (substitutions); black dots represent hypothetical haplotypes. Red borders denote Arc1 haplotypes, orange borders denote Arc2 haplotypes. Black borders denote haplotypes carried by dingoes; the central haplotype is A29. Blue filling denotes haplotypes found in the specific region and shared with other regions in the Old World; yellow filling denotes haplotypes unique to the region; no filling denotes haplotypes not present but found in other regions; green filling denotes the New Guinean haplotype A79.

We analysed 582 bp of the mtDNA control region (nucleotide positions 15 458–16 039 of the mitochondrial genome) in a total sample of 674 dogs, 232 dingoes and 3 NGSDs (electronic supplementary material, file S1). In the present study, we sequenced 305 samples of dog (84 from Mainland Southeast Asia and 221 from across Island Southeast Asia), 21 dingoes and 1 NGSD (all novel haplotypes have been deposited in GenBank under accession numbers HQ452433 and HQ452439–HQ452465), and from previous studies we included sequences from 369 dogs (281 from South China, 59 from Mainland Southeast Asia, 10 from Island Southeast Asia and 19 from Polynesia), 211 dingoes and 2 NGSDs [9,35,37]. The 19 samples from Polynesia were archaeological specimens and analysed for a shorter region (263 bp; positions 15 458–15 720). These data were also compared with 1224 sequences from dogs throughout the world [9], for creating minimum spanning networks based on a global dog sample (figure 1). The geographical distribution of samples specifically studied are as follows. Australia (dingo, *n* = 232): Northern Territory (*n* = 3), Queensland (*n* = 44), Western Australia (*n* = 29), South Australia (*n* = 6), Victoria (*n* = 35), New South Wales (*n* = 110) and miscellaneous (*n* = 5). South China (*n* = 281): Guangdong (*n* = 14), Guangxi (*n* = 35), Guizhou (*n* = 57), Hunan (*n* = 54), Jiangxi (*n* = 46) and Yunnan (*n* = 75). Southeast Asia (*n* = 143): Cambodia (*n* = 8), Thailand (*n* = 105) and Vietnam (*n* = 30). Indonesia (*n* = 131): Bali (*n* = 61 of which Datah, *n* = 2; Lembongan island, *n* = 37; Tenganan, *n* = 22), Kalimantan (*n* = 65 of which Latta Laga, *n* = 43; Loksad, *n* = 12; Mallinau, *n* = 8; miscellaneous, *n* = 2), Sulawesi (*n* = 3) and miscellaneous (*n* = 2). New Guinea (*n* = 15): New Guinea Highland (NGSD, *n* = 3) and New Guinea Lowland (dog; *n* = 12). Taiwan (*n* = 52): Wufeng (*n* = 12), Jen'al (*n* = 15), Mawlin (*n* = 9), Mutai (*n* = 3), Shiowlin (*n* = 2) and miscellaneous (*n* = 11). The Philippines: (*n* = 36). Additionally, from Polynesia, 19 pre-European archaeological samples were analysed: Cook Islands (*n* = 2), Hawaii (*n* = 4) and New Zealand (*n* = 13). The Taiwanese dog samples were collected from the Austronesian-speaking peoples Atayal, Seedeq and Rukai, the samples from Kalimantan from the Austronesian-speaking people Dayak, and the samples from Bali from the Austronesian-speaking Balinese people, while the samples from South China, Southeast Asia and the Philippines were collected mostly from rural locations. The samples were collected to avoid crossbreeding with modern breed dogs and relatedness among individuals: the dogs were not stray (all had an owner) and were sampled in areas with a low influx of foreign dogs (not more than one individual per family or pack).

### (b) DNA extraction, amplification and sequencing

The samples were collected as Heparin-treated blood samples (174 samples, 165 of which transferred to FTA cards; FTA, fast technology for analysis of nucleic acids), 119 buccal cell samples on FTA cards, 22 hair samples and 12 modern skull samples (collected in 1981). Hair samples were extracted according to Angleby & Savolainen [38]; Heparin-treated blood samples, buccal epithelial cell samples on Whatman FTA cards and Heparin-treated blood samples transferred to FTA cards were extracted as by Natanaelsson *et al*. [39]; and skull samples as by Elledge *et al*. [40]. PCR amplification was performed as described by Angleby & Savolainen [38]. DNA sequencing was performed in forward and reverse directions over all nucleotide positions, using ABI Big Dye terminator chemistry and analysis on ABI 3700 DNA sequencer as described by Angleby & Savolainen [38].

### (c) Analysis of sequence data

The DNA sequences were edited using Sequencing Analysis (Applied Biosystems) and assembled into contigs and further edited using Sequencher v. 4.1 (Gene Codes Corporation).

To display the phylogenetic relations between haplotypes, minimum spanning networks were created by calculating distances using Arlequin v. 3.11 software [41] and then drawn by hand. The networks were based on the sequences from the dogs, dingoes and NGSDs from the specifically studied geographical region, as well as from 1224 dogs from across the world [9], to display the global phylogeny of dogs.

The mutation rate for the 582 bp region was obtained from Pang *et al*. [9], where the average genetic distance between dog/wolf and coyote in a phylogenetic tree was calibrated with the time for the separation between the wolf and coyote lineages. There is no exact calibration point for the wolf–coyote separation, but a possible range of 1.5–4.5 Ma. This gives a rate of 1.1 × 10^−8^–4.3 × 10^−8^ substitutions per year, or 1 substitution per 40 000–155 000 years (the rate in [9] is given as substitutions per site per year, but should be substitutions per year).

The time of arrival of dingoes to Australia was estimated using the statistic *ρ* (the mean number of substitutions for a set of sequences to their common ancestral haplotype) [42], calculating the mean distance to haplotype A29 for the dingo sequences, and the substitution rate. Because of the range of possible separation times between wolf and coyote, and of the resultant substitution rate, the time estimate is also obtained as a relatively broad range of possible time. The standard error for *ρ* was calculated by resampling, with the size of the original number of individuals, in 1000 replicates using the program avdist (Lars Arvestad).

## 3. Results

We compared haplotypes detected in dog samples from South China, Mainland Southeast Asia, Indonesia, New Guinea, the Philippines and Taiwan with those previously identified among ancient, pre-European samples from Polynesia and the Australian dingo and NGSD populations [9,35,37]. Together with the pre-European Polynesian samples, dogs of Austronesian-speaking populations across most of Island Southeast Asia and Oceania were covered. The haplotypes detected in the different regions are indicated in a minimum spanning network (figure 1; see electronic supplementary material, files S1 and S2 for detailed information). The network was constructed based on the 909 samples from the studied geographical region as well as the 1224 samples of dog from across the world [9], putting the regional haplotypes into the context of global dog diversity. Only the major dog haplogroup, clade A [9], is shown since the haplotypes found among the Polynesian dogs and Australian dingoes fall only into this haplogroup [35].

**Figure 2.**
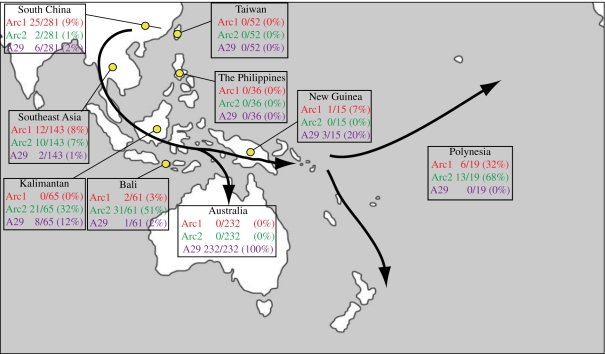
Frequency of the Polynesian haplotypes Arc1 and Arc2, and the dingo founder haplotype A29 in geographical regions. The number of individuals carrying each haplotype, total number of samples for the region and frequency (per cent) are shown. Arrows indicate suggested introduction routes. For Australia, A29 denotes both haplotypes A29 and A29′ (see text).

Among the 19 pre-European samples from Polynesia two haplotypes were detected, Arc1 (found in 6 of the 19 samples, 32%) and Arc2 (13/19, 68%; figures 1 and 2). Both haplotypes were found at all three sampled locations, situated across large parts of Polynesia: Cook Islands (Arc1 *n* = 1; Arc2 *n* = 1), Hawaii (Arc1 *n* = 2; Arc2 *n* = 2) and New Zealand (Arc1 *n* = 3; Arc2 *n* = 10). Since the samples are from archaeological remains, they were analysed only for a shorter stretch (263 bp), and therefore correspond to several different full-length haplotypes (13 and 2, respectively; figure 1). Among the Australian dingoes, all 232 samples (100%) had either haploptype A29 or a haplotype separated from A29 by one substitutional step (designated A29′). Haplotype A29 is shared with domestic dogs, while 11 of the A29′ haplotypes are unique to the dingo population and a 12th shared with dogs (an Arc1 haplotype, but found in a single individual and probably formed by a back mutation, and therefore treated as A29′). The sample from New Guinea consisted of three NGSDs, representing the only three known female lineages, and 12 dogs from lowland villages. Two NGSDs had haplotype A29 and one had haplotype A79, which is separated from A29 by one substitutional step, indicating a common ancestry with dingoes. Among the village dogs, two had A79 (unique to NGSDs and New Guinea lowland village dogs), and A29 and Arc1 were each detected in one individual.

South China is the probable source population for the studied region, and in accordance with this we detected both the two Polynesian haplotypes and the dingo founder type, A29, in this sample. Arc1 was found in 25 of the 281 samples (9%), Arc2 in two dogs (1%) and A29 in six dogs (2%). Among the 281 samples (collected from Guangdong, Guangxi, Guizhou, Hunan, Jiangxi and Yunnan), 69 haplotypes were detected in total, 34 of which were unique to the region.

In Mainland Southeast Asia, 51 haplotypes were detected, 18 of which were unique, among 143 samples. Both Polynesian haplotypes and A29 were found here also, at similar frequency to that in South China: Arc1 in 13 of 143 samples (9%), Arc2 in 10 samples (7%) and A29 in two samples (1%).

In total, 131 samples from Indonesia were analysed, and 23 haplotypes detected, eight of which were unique to the region. Both of the Polynesian haplotypes, and the dingo haplotype A29, were detected: Arc1 in two of 131 samples (2%), Arc2 in 53 samples (40%) and A29 in 10 samples (8%). The two most common haplotypes in the region were A75 (which is one of the two possible full-length haplotypes of the Polynesian haplotype Arc2), carried by 52 dogs (40%), and the dingo founder haplotype A29, carried by 10 dogs (8%). Most of the Indonesian samples were from Bali (*n* = 61) and Kalimantan (*n* = 65); Bali had 3 per cent Arc1, 51 per cent Arc2 and 2 per cent A29, and Kalimantan had 0 per cent Arc1, 32 per cent Arc2 and 12 per cent A29. Surrounding haplotype Arc2 (corresponding to full-length haplotypes A75 and A120) in the minimum spanning network were haplotype A195 (unique in Indonesia) and A145 (shared between China and Indonesia but not detected elsewhere). The haplotypes in this clade were represented in 59 dogs (45%) in the Indonesian sample.

Among the 52 Taiwanese samples, 17 haplotypes were found, two of which were unique, but none of A29, Arc1 or Arc2 were represented. Similarly, among the 36 samples from the Philippines we detected 17 haplotypes, three of which were unique to the Philippines, but none of Arc1, Arc2 or A29 was represented.

To summarize, the two mtDNA haplotypes Arc1 and Arc2 were found in 100 per cent of Polynesian ancient dog samples, and A29 and A29′ were found in 100 per cent of investigated dingoes and NGSDs. Arc1 and Arc2 were also carried by 10 per cent of the dogs in South China, by 16 per cent in Mainland Southeast Asia and by 42 per cent in Indonesia, but were absent in the samples from Taiwan and the Philippines. Similarly, A29 had a frequency of 2 per cent in South China, 1 per cent in Mainland Southeast Asia, 8 per cent in Indonesia, but 0 per cent in Taiwan and the Philippines. The probability that the three haplotypes would be present in the Taiwanese or Philippine populations but not sampled in our study is low. For example, if the frequencies for Taiwan are assumed to be the same as in South China (which had the lowest frequencies in the studied region, except Taiwan and the Philippines), the probability of not finding any of the three haplotypes among 52 samples is 0.0015.

Based on the assumption that A29 was the only haplotype carried by the dingo founders, the introduction of dingoes to Australia has previously been dated to approximately 5000 years BP, and possibly up to 10 000 years BP, years BP [35], from the mean genetic distance among dingo sequences (*ρ*) to A29 [42]. Based on the present sample of dingoes, with *ρ* = 0.116 (s.e. = 0.0005), excluding 25 samples from Pilbarra in Western Australia probably affected by genetic drift [35] and using a conservative recalculation of the mutation rate [9], we estimate the time of arrival of dingoes to 4640–18 100 years BP (4600–18 300, 95% confidence limits). These calculations are dependent on A29 being the only founder; if any of the other haplotypes found among the dingoes was also introduced from outside Australia, an underestimation of the time of arrival is obtained. However, since all other haplotypes but one (carried by a single individual and possibly formed by a back mutation) were unique to Australia, the assumption that A29 was the only founder haplotype seems reasonable.

## 4. Discussion

This study shows a distinct pattern in the geographical distribution of the two Polynesian dog mtDNA haplotypes Arc1 and Arc2, and the dingo and NGSD founder mtDNA haplotype A29, with a total frequency of 12 per cent in Southern China, 17 per cent in southeast Asia and 50 per cent in Indonesia, but complete absence in samples from Taiwan and the Philippines. This gives a clear indication that Polynesian dogs as well as dingoes and NGSDs trace their ancestry back to South China through Mainland Southeast Asia and Indonesia. Thus, there is no indication that these dogs were introduced via Taiwan and the Philippines together with the expansion of the Neolithic culture and Austronesian languages, as suggested in some theories about Polynesian origins.

The phylogeographic pattern for the dogs, with the two Polynesian haplotypes traceable only through Indonesia and southeast Asia, is mirrored by that of pigs, for which mtDNA haplotypes belonging to a ‘Pacific clade’ were found in pigs from Polynesia, Indonesia and Southeast Asia, but absent among pigs from Taiwan and the Philippines [43]. Thus, while this study has no direct bearing on the origin of the Polynesian people it indicates that, in the case they trace their origin from Taiwan, their Neolithic cultural package was modified en route; at least the domesticated dog and pig seem to have spread from Mainland Southeast Asia to Indonesia and Melanesia, where they were picked up by the Polynesian ancestors. A presence of dogs in Island Southeast Asia before the arrival of the Neolithic from Taiwan is also indicated by the mtDNA-based dating of the arrival of dingoes to Australia, greater than 4600 BP.

Therefore, among the different models for the origins of the Polynesians and their culture, the dog mtDNA data do not support the more extreme express train model, which suggests a rapid spread of farmers from South China via Taiwan, with little cultural exchange between farmers and the indigenous populations in eastern Indonesia and Melanesia. Instead, the data fit better with models proposing an extensive interaction between indigenous Indonesians/Melanesians and intruding farmers (e.g. the triple I model) [19]. Both the archaeological culture and the language of the Polynesians are clearly indicated to have originated from Taiwan, but genetic studies of humans have indicated that only a fraction of the Polynesian mtDNA and Y chromosome gene pools originated from Taiwan. We suggest that, with the evidence on the origins of Polynesian domestic dogs and pigs, a likely scenario for the origins of Polynesians is that farmers spread from Taiwan bringing the Neolithic culture (e.g. pottery) and Austronesian languages, but mixed extensively with local Melanesian populations, and picked up some cultural traits (e.g. the domesticated dog and pig, and the commensal Polynesian rat [44]) en route. Therefore, the cultural package of the Polynesians was probably formed from different sources, some parts deriving from Taiwan and others incorporated at the spread through Indonesia and Melanesia.

Earlier studies have shown the feral Australian dingo to have an ultimate origin from East Asian domestic dogs [35]. The possibility that the dingo was introduced to Australia from Taiwan in connection with the Austronesian expansion has been discussed [35], but the distribution of haplotype A29 indicates that the introduction route was instead through Mainland Southeast Asia. Importantly, except for the possibly Neolithic-related dingoes, no clear signs of Neolithic culture have been observed in prehistoric Australia. How the dingoes were introduced is therefore unclear. It may have happened by way of limited contacts with Neolithic groups (e.g. through trading with Austronesians in New Guinea, something that has been practiced in historical times via the Torres straits [6]) or it may have happened through contact with pre-Neolithic groups. Importantly, NGSDs, from the pre-Neolithic New Guinean highlands, carried only haplotypes A29 (the dingo founder haplotype) and A79 (separated from A29 by one substitutional step). This strongly indicates a common origin of these two ‘pre-Neolithic’ dog populations. A common ancestry of NGSDs and dingoes is also suggested by similarities in morphology and behaviour [36], as well as sharing of dog leukocyte antigen (DLA) haplotypes [45]. Among the 12 samples from lowland New Guinea, one had haplotype A29 and two had A79 (unique to NGSDs and the lowland village dogs), indicating a relation between the lowland and highland populations.

The earliest archaeological evidence for dingoes in Australia has been dated to 3500 years BP [1,2], but, based on the mtDNA data, we estimate the time of arrival of dingoes to 4600–18 300 years BP. Thus, the genetic data suggest a date that is considerably earlier than suggested by the archaeological evidence, and earlier than the arrival of the Neolithic to the surrounding regions (e.g. Lapita). Therefore, dingoes possibly arrived through contacts with pre-Neolithic populations, and do not constitute a sign of contact with the Neolithic. Dogs are generally believed to have originated before the Neolithic [9], and were possibly introduced to Mainland and Island Southeast Asia earlier than indicated by the sparse archaeological record, as a truly pre-Neolithic domesticate, before being included in the Austronesian Neolithic cultural package.

Australian dingoes and NGSDs carried only haplotypes A29 and A29′, and Polynesian domestic dogs carried two haplotypes, Arc1 and Arc2. Thus, it seems that only a single mtDNA haplotype was introduced to Australia and two others to Polynesia. However, this does not necessarily imply that dingoes and Polynesian dogs did not originate from the same population. All three haplotypes are found in Mainland and Island Southeast Asia, among several other haplotypes. It is possible that the dingo and Polynesian dog populations were founded from very few individuals from the same Indonesian population, but obtained different haplotypes because of founder bottlenecks.

## 5. Conclusions

There is archaeological evidence that dogs had spread across Mainland and Island Southeast Asia, and to Australia, by 3000–4000 years BP, possibly in parallel with a Neolithic farming expansion. It has been assumed that the Polynesian culture, including the domesticated dog, pig and chicken, spread from Taiwan in connection with the Austronesian expansion, and that the dingo may also have been introduced in this context. However, the data presented here indicate that the Polynesian domestic dogs trace their ancestry from Mainland Southeast Asia, and that dogs may have been present in Island Southeast Asia before the arrival of the Neolithic. Therefore, the Polynesian culture probably had a complex origin, with components from Taiwan as well as Indonesia and Melanesia. For the Australian dingoes and the NGSDs also, the likely introduction route was through Mainland Southeast Asia, possibly in connection with the Polynesian dogs. The mtDNA data suggest that dingoes arrived earlier than indicated by the archaeological record, before the arrival of the Neolithic to the surrounding regions. Whether the dingo was actually a Neolithic item that spread to an Australian continent otherwise unaffected by the Neolithic, or was introduced as a pre-Neolithic domesticate, remains to be elucidated.

## References

[1] Milham P. Thompson P. 1976 Relative antiquity of human occupation and extinct fauna at Madura cave, Southeastern Western Australia. Mankind 10, 175–180 10.1111/j.1835-9310.1976.tb01149.x (doi:10.1111/j.1835-9310.1976.tb01149.x)

[2] Gollan K. 1984 The Australian dingo: in the shadow of man. In Vertebrate zoogeography and evolution in Australasia (eds Archer M. Clayton G.), pp. 921–927 Carlisle, Australia: Hesperian Press

[3] Kirch P. V. Weisler M. I. 1994 Archaeology in the Pacific Islands: an appraisal of recent research. J. Archaeol. Res. 2, 285–328 10.1007/BF02231482 (doi:10.1007/BF02231482)

[4] Kirch P. V. 1997 The Lapita people: ancestors of the Oceanic world. Oxford, UK: Blackwell Publishing

[5] Bellwood P. 2005 First farmers: the origins of agricultural societies. Oxford, UK: Blackwell Publishing

[6] Mulvaney J. Kamminga J. 1999 Prehistory of Australia. Washington, DC: Smithsonian Institution Press

[7] Wickler S. Spriggs M. 1988 Pleistocene human occupation of the Solomon Islands, Melanesia. Antiquity 62, 703–706

[8] Spriggs M. Anderson A. 1993 Late colonization of East Polynesia. Antiquity 67, 200–217

[9] Pang J.-F. 2009 mtDNA data indicate a single origin for dogs South of Yangtze River, less than 16 300 years ago, from numerous wolves. Mol. Biol. Evol. 26, 2849–2864 10.1093/molbev/msp195 (doi:10.1093/molbev/msp195) 19723671 PMC2775109

[10] Higham C. F. W. 1996 A review of archaeology in Mainland Southeast Asia. J. Arch. Res. 7, 149–165 10.1007/BF02228837 (doi:10.1007/BF02228837)

[11] Bellwood P. 1997 Prehistory of the Indo-Malaysian archipelago. Honolulu, HI: University of Hawaii Press

[12] Bellwood P. White P. 2005 Domesticated pigs in Eastern Indonesia. Science 309, 381 10.1126/science.309.5733.381a (doi:10.1126/science.309.5733.381a) 16020714

[13] Matisoo-Smith E. 2007 Animal translocations, genetic variation and the human settlement of the Pacific. In Genes, language and culture history in the southwest Pacific (ed. Friedlaender J. S.), pp. 157–170 Oxford, UK: Oxford University Press

[14] Hurles M. E. Matisoo-Smith E. Gray R. D. Penny D. 2003 Untangling oceanic settlement: the edge of the knowable. Trends Ecol. Evol. 18, 531–540 10.1016/S0169-5347(03)00245-3 (doi:10.1016/S0169-5347(03)00245-3)

[15] Diamond J. M. 1988 Express train to Polynesia. Nature 336, 307–308 10.1038/336307a0 (doi:10.1038/336307a0)

[16] Diamond J. 2001 Polynesian origins: slow boat to Melanesia? (Reply to: S. Oppenheimer & M. Richards). Nature 410, 167 10.1038/35065523 (doi:10.1038/35065523) 11242066

[17] Terrell J. E. 1988 History as a family tree, history as an entangled bank: constructing images and interpretations of prehistory in the South Pacific. Antiquity 62, 642–657

[18] Terrell J. E. Kelly K. M. Rainbird P. 2001 Foregone conclusions? In search of ‘Papuans’ and ‘Austronesians’. Curr. Anthropol. 42, 97–124 10.1086/318436 (doi:10.1086/318436)

[19] Green R. C. 1991 The Lapita cultural complex: current evidence and proposed models. Indo-Pacific Prehistory Assoc. Bull. 11, 295–305

[20] Bellwood P. 1987 The prehistory of Island Southeast Asia: a multidisciplinary review of recent research. J. World Prehistory 1, 171–224 10.1007/BF00975493 (doi:10.1007/BF00975493)

[21] Blust R. 1995 The prehistory of the Austronesian-speaking peoples: a view from language. J. World Prehistory 9, 453–510 10.1007/BF02221119 (doi:10.1007/BF02221119)

[22] Gray R. D. Drummond A. J. Greenhill S. J. 2009 Language phylogenies reveal expansion pulses and pauses in Pacific settlement. Science 323, 479–483 10.1126/science.1166858 (doi:10.1126/science.1166858) 19164742

[23] Greenhill S. J. Drummond A. J. Gray R. D. 2010 How accurate and robust are the phylogenetic estimates of Austronesian language relationships? PLoS ONE 5, e9573 10.1371/journal.pone.0009573 (doi:10.1371/journal.pone.0009573) 20224774 PMC2835747

[24] Kayser M. Brauer S. Weiss G. Underhill P. A. Roewer L. Schiefenhövel W. Stoneking M. 2000 Melanesian origin of Polynesian Y chromosomes. Curr. Biol. 10, 1237–1246 10.1016/S0960-9822(00)00734-X (doi:10.1016/S0960-9822(00)00734-X) 11069104

[25] Oppenheimer S. J. Richards M. 2001 Slow boat to Melanesia? Nature 410, 166–167 10.1038/35065520 (doi:10.1038/35065520) 11242066

[26] Redd A. J. Takezaki N. Sherry S. T. McGarvey S. T. Sofro A. S. M. Stoneking M. 1995 Evolutionary history of the COII/tRNA^Lys^ intergenic 9 base pair deletion in human mitochondrial DNAs from the Pacific. Mol. Biol. Evol. 12, 604–615 7659016 10.1093/oxfordjournals.molbev.a040240

[27] Hagelberg E. Kayser M. Nagy M. Roewer L. Zimdahl H. Krawczak M. Lió P. Schiefenhövel W. 1999 Molecular genetic evidence for the human settlement of the Pacific: analysis of mitochondrial DNA, Y chromosome and HLA markers. Phil. Trans. R. Soc. Lond. B 354, 141–152 10.1098/rstb.1999.0367 (doi:10.1098/rstb.1999.0367) 10091254 PMC1692446

[28] Soares P. 2011 Ancient voyaging and Polynesian origins. Am. J. Hum. Genet. 88, 1–9 10.1016/j.ajhg.2011.01.009 (doi:10.1016/j.ajhg.2011.01.009) PMC3035714 21295281

[29] Capelli C. 2001 A predominantly indigenous paternal heritage for the Austronesian-speaking peoples of insular southeast Asia and Oceania. Am. J. Hum. Genet. 68, 432–443 10.1086/318205 (doi:10.1086/318205) 11170891 PMC1235276

[30] Hurles M. E. Nicholson J. Bosch E. Renfrew C. Sykes B. C. Jobling M. A. 2002 Y chromosomal evidence for the origins of Oceanic-speaking peoples. Genetics 160, 289–303 11805064 10.1093/genetics/160.1.289 PMC1461928

[31] Kayser M. 2006 Melanesian and Asian origins of Polynesians: mtDNA and Y chromosome gradients across the Pacific. Mol. Biol. Evol. 23, 2234–2244 10.1093/molbev/msl093 (doi:10.1093/molbev/msl093) 16923821

[32] Kayser M. Choi Y. van Oven M. Mona S. Brauer S. Trent R. J. Suarkia D. Schiefenhövel W. Stoneking M. 2008 The impact of the Austronesian expansion: evidence from mtDNA and Y chromosome diversity in the Admiralty islands of Melanesia. Mol. Biol. Evol. 25, 1362–1374 10.1093/molbev/msn078 (doi:10.1093/molbev/msn078) 18390477

[33] Hudjashov G. 2007 Revealing the prehistoric settlement of Australia by Y chromosome and mtDNA analysis. Proc. Natl Acad. Sci. USA 104, 8726–8730 10.1073/pnas.0702928104 (doi:10.1073/pnas.0702928104) 17496137 PMC1885570

[34] McEvoy B. P. Lind J. M. Wang E. T. Moyzis R. K. Visscher P. M. van Holst Pellekaan S. M. Wilton A. N. 2010 Whole-genome genetic diversity in a sample of Australians with deep aboriginal ancestry. Am. J. Hum. Genet. 87, 297–305 10.1016/j.ajhg.2010.07.008 (doi:10.1016/j.ajhg.2010.07.008) 20691402 PMC2917718

[35] Savolainen P. Leitner T. Wilton A. N. Matisoo-Smith E. Lundeberg J. 2004 A detailed picture of the origin of the Australian dingo, obtained from the study of mitochondrial DNA. Proc. Natl Acad. Sci. USA 101, 12 387–12 390 10.1073/pnas.0401814101 (doi:10.1073/pnas.0401814101) PMC514485 15299143

[36] Koler-Matznick J. Lehr Brisbin I. Jr Feinstein M. Bulmer S. 2003 An updated description of the New Guinea Singing Dog (*Canis hallstromi*, Troughton 1957). J. Zool. Lond. 261, 109–118 10.1017/S0952836903004060 (doi:10.1017/S0952836903004060)

[37] Savolainen P. Zhang Y.-P. Luo J. Lundeberg J. Leitner T. 2002 Genetic evidence for an East Asian origin of domestic dogs. Science 298, 1610–1613 10.1126/science.1073906 (doi:10.1126/science.1073906) 12446907

[38] Angleby H. Savolainen P. 2004 Forensic informativity of domestic dog mtDNA control region sequences. Forensic Sci. 154, 99–110 10.1016/j.forsciint.2004.09.132 (doi:10.1016/j.forsciint.2004.09.132) 16182956

[39] Natanaelsson C. Oskarsson M. C. R. Angleby H. Lundeberg J. Kirkness E. Savolainen P. 2006 Dog Y chromosomal DNA sequence: identification, sequencing and SNP discovery. BMC Genet. 7, 45 10.1186/1471-2156-7-45 (doi:10.1186/1471-2156-7-45) 17026745 PMC1630699

[40] Elledge A. E. Allen L. R. Carlsson B.-L. Wilton A. N. Leung P. 2008 An evaluation of genetic analyses, skull morphology and visual appearance for assessing dingo purity: implications for dingo conservation. Wildl. Res. 35, 812–820 10.1071/WR07056 (doi:10.1071/WR07056)

[41] Excoffier L. Laval G. Schneider S. 2005 Arlequin (version 3.0): an integrated software package for population genetics data analysis. Evol. Bioinf. Online 1, 47–50 PMC2658868 19325852

[42] Forster P. Harding R. Torroni A. Bandelt J. 1996 Origin and evolution of Native American mtDNA variation: a reappraisal. Am. J. Hum. Genet. 59, 935–945 8808611 PMC1914796

[43] Larson G. 2007 Phylogeny and ancient DNA of *Sus* provides insights into Neolithic expansion in Island Southeast Asia and Oceania. Proc. Natl Acad. Sci. USA 104, 4834–4839 10.1073/pnas.0607753104 (doi:10.1073/pnas.0607753104) 17360400 PMC1829225

[44] Matisoo-Smith E. Robins J. H. 2004 Origins and dispersals of Pacific peoples: evidence from mtDNA phylogenies of the Pacific rat. Proc. Natl Acad. Sci. USA 101, 9167–9172 10.1073/pnas.0403120101 (doi:10.1073/pnas.0403120101) 15184658 PMC428491

[45] Runstadler J. A. Angles J. M. Pedersen N. C. 2006 Dog leucocyte antigen class II diversity and relationships among indigenous dogs of the island nations of Indonesia (Bali), Australia and New Guinea. Tissue Antigens 68, 418–426 10.1111/j.1399-0039.2006.00696.x (doi:10.1111/j.1399-0039.2006.00696.x) 17092255

